# Eupalinolide B Alleviates Oxidative Stress in LPS-Induced RAW264.7 Macrophages via Covalently Binding to PRDX4

**DOI:** 10.3390/biomedicines14030629

**Published:** 2026-03-11

**Authors:** Ruishen Zhuge, Jianru Liu, Yueming Tian, Lirun Zhou, Yuanbo Wang, Huan Tang, Jinsheng Zhong, Wenhua Kuang, Xiangying Ouyang

**Affiliations:** 1 Department of Periodontology, Peking University School and Hospital of Stomatology, National Center for Stomatology, National Clinical Research Center for Oral Diseases, National Engineering Research Center of Oral Biomaterials and Digital Medical Devices, Beijing 100081, China; zhugedentist@pku.edu.cn (R.Z.); lensang@163.com (J.L.); 18811585677@163.com (Y.W.); zhongjs2012@163.com (J.Z.); 2 Department of Mechanical Engineering, Tsinghua University, Beijing 100084, China; tym00007@163.com; 3 State Key Laboratory for Quality Ensurance and Sustainable Use of Dao-Di Herbs, Artemisinin Research Center, Institute of Chinese Materia Medica, China Academy of Chinese Medical Sciences, Beijing 100700, China; lirunzhou1997@163.com (L.Z.); huant@icmm.ac.cn (H.T.)

**Keywords:** Eupalinolide B, oxidative stress, chemical proteomics, target identification, PRDX4

## Abstract

**Background/Objectives:** Eupalinolide B (EB), a natural compound derived from *Eupatorium lindleyanum* DC, has demonstrated multiple pharmacological activities. However, its role in modulating oxidative stress remains incompletely understood. **Methods:** In this study, we investigated the antioxidant effect and underlying mechanism of EB in lipopolysaccharide (LPS)-induced RAW264.7 macrophages. **Results:** EB significantly attenuated LPS-induced oxidative stress as evidenced by reduced levels of intracellular reactive oxygen species (ROS), nitric oxide (NO), and malondialdehyde (MDA) alongside enhanced superoxide dismutase (SOD) activity and an increased reduced/oxidized glutathione (GSH/GSSG) ratio. Using activity-based protein profiling, we identified peroxiredoxin 4 (PRDX4) as a key binding target of EB. Direct interaction was confirmed through labeling and competitive binding assays with purified PRDX4 protein. High-resolution mass spectrometry revealed that EB covalently binds to Cys54 and Cys248 residues of PRDX4. Furthermore, EB treatment upregulated PRDX4 protein expression in LPS-stimulated RAW264.7 cells. siRNA-mediated knockdown of PRDX4 significantly blunted the antioxidant effects of EB, confirming the functional relevance of this target. **Conclusions:** Our findings demonstrate that EB alleviates LPS-induced oxidative stress in macrophages by covalently binding to and stabilizing PRDX4, thereby enhancing cellular antioxidant capacity. This study unveils a novel mechanism whereby a natural product enhances cellular antioxidant capacity by covalently stabilizing a key peroxidase, highlighting the potential of EB as a therapeutic agent and PRDX4 as a promising target for oxidative stress-related diseases.

## 1. Introduction

The mutual amplification between oxidative stress and inflammation represents a core pathological mechanism underlying numerous chronic diseases [1,2]. Consequently, therapeutic strategies aimed at enhancing antioxidant capacity have emerged as a pivotal direction for future disease treatment [3,4]. As key cells of the innate immune system, macrophages are the central regulators of inflammatory responses and oxidative stress [5]. Under pathological conditions such as infection and tissue injury, macrophages are overactivated and secrete large amounts of reactive oxygen species (ROS) and nitric oxide (NO), a major reactive nitrogen species (RNS)—along with inflammatory cytokines (e.g., TNF-α, IL-6, and IL-1β)—which constitute a common pathological driver of inflammation-related diseases [6,7,8,9,10]. Notably, while NO functions as a physiological signaling molecule (e.g., mediating vascular relaxation) at basal concentrations, its pathological overproduction such as when induced by lipopolysaccharide (LPS) synergizes with excess ROS to form peroxynitrite, a highly oxidizing RNS derivative that exacerbates oxidative damage (e.g., elevated malondialdehyde (MDA) a marker of lipid peroxidation) [11]. The resulting oxidative damage extends to the impairment of the multilayered antioxidant defense system, characterized by diminished activity of enzymatic antioxidants such as superoxide dismutase (SOD) and a disturbed equilibrium of the non-enzymatic glutathione (GSH/GSSG) redox buffer [12,13].

The antioxidant defense system relies on coordinated interactions between enzymatic and non-enzymatic components to maintain redox homeostasis, which represents a dynamic state, not a static binary equilibrium [14]. For example, SOD acts as a frontline enzyme converting superoxide anions, a major ROS to H_2_O_2_, which is further scavenged by downstream enzymatic systems (e.g., glutathione peroxidase, GPx) or directly decomposed—processes that often depend on GSH as a key reducing cofactor [15,16]. Notably, this multi-layered defense network also relies on an essential yet often underemphasized family of peroxidases—peroxiredoxins (PRDXs) [17]. Peroxiredoxin 4 (PRDX4), an endogenous intracellular antioxidant enzyme belonging to the peroxiredoxin family, functions as a typical 2-cysteine (2-Cys) peroxiredoxin [18,19]. PRDx4 not only efficiently removes peroxides such as H_2_O_2_ and lipid peroxides by using glutathione and thioredoxin as electron donors, but also maintains the homeostasis of disulfide bonds in the endoplasmic reticulum [20]. PRDX4 can work in synergy with classical antioxidant enzymes to enhance the stability of the entire antioxidant network [18]. Accumulating evidence indicates that PRDX4 dysfunction induces oxidative damage and endoplasmic reticulum stress, contributing to the pathogenesis of various diseases, including diabetes, arthritis and cancer [21,22,23]. These findings collectively highlight PRDX4 as a promising therapeutic target for modulating oxidative stress.

Traditional Chinese medicinal herbs have garnered considerable attention as a valuable source of natural antioxidants [24]. In recent years, natural products have become a focal point in antioxidant drug discovery, owing to their advantages such as fewer side effects and lower propensity for drug resistance compared to conventional synthetic compounds [25]. However, the pharmacological actions of many natural products, including those derived from Chinese medicine, often exhibit multi-target and multi-pathway characteristics, which complicate the precise elucidation of their mechanisms of action [26,27]. Therefore, identifying the specific protein targets through which natural products exert their distinct pharmacological effects remains a critical and challenging task. Activity-based protein profiling (ABPP) has effectively overcome the limitations of traditional transcriptomic and genomic approaches, such as their narrow applicability and susceptibility to interference [28,29,30]. Eupalinolide B is a principal bioactive constituent isolated from Eupatorium lindleyanum DC and displayed a spectrum of pharmacological activities associated with inflammatory conditions such as bronchitis, cough, and asthma due to the α-β unsaturated aldehyde-ketone structure, which can covalently bind to the active sites of various proteins [31,32]. Despite significant progress in elucidating the multifaceted bioactivities of EB, the specific protein targets and molecular pathways underlying its antioxidant effects remain unclear.

In this study, we employed an activity-based protein profiling (ABPP) strategy to identify the direct cellular targets of EB in lipopolysaccharide (LPS)-stimulated RAW264.7 macrophages—a well-established model of inflammation-associated oxidative stress [33,34]. This study discovered for the first time that EB forms a covalent bond at the cysteine 54/248 site with PRDX4, enhancing the post-translational stability of PRDX4 without altering its activity/transcription to improve total antioxidant capacity in cells. This reveals a new natural product action mode (covalent peroxide reductase stabilization) and provides guidance for the development of future redox regulators targeting PRDX4.

## 2. Materials and Methods

### 2.1. Agents and Chemicals

The supplier of cycloheximide (CHX) (98% pure) was commercially acquired from Herb Purify Co., Ltd. (Chengdu, China).

### 2.2. Cell Line Selection and Culture

RAW264.7 cells were cultured in high-glucose DMEM medium (Corning, NY, USA, Catalog No.: C11995500BT, Gibco) supplemented with 10% fetal bovine serum (FBS, Catalog No.: 10099141C, Gibco, Corning, NY, USA). The cells were maintained at 37 °C in a humidified atmosphere containing 5% CO_2_. To induce oxidative stress, RAW264.7 cells were stimulated with 1 μg/mL LPS (Sigma-Aldrich, St. Louis, MO, USA, 297-473-0) and exposed to EB or the EB fluorescent probe (EB-P) (Shanghai Standard BioResearch Co., Ltd., Shanghai, China, ST04620120) for durations ranging from 0 to 24 h.

### 2.3. Detection of Intracellular Reactive Oxygen Species Content

The cell culture medium was removed and an appropriate volume of diluted 2′,7′-dichlorofluorescin (DCFH-DA) (Beyotime, Nantong, China, S0033S) was added to achieve a final concentration of 10 μmol/L. The volume of diluted DCFH-DA was adjusted to ensure complete coverage of the cells with a minimum addition of 1 mL per well in six-well plates. Cells were incubated in a 37 °C cell incubator for 20 min. Subsequently, the cells were washed three times with Opti-MEM (Thermo Fisher Scientific, Waltham, MA, USA) to completely remove the uninternalized DCFH-DA. After trypsin digestion, fluorescence intensity was quantified using a BD FACSCanto II flow cytometer (BD Biosciences, Faucett, NJ, USA) or imaged via an Olympus IX73 fluorescence microscope (Olympus Corporation, Tokyo, Japan). The average fluorescence intensity of cells was calculated from three independent fields using the Image J software (v1.53, NIH, Bethesda, MD, USA).

### 2.4. Detection of Intracellular Antioxidant Index

After treatment, cells were rinsed once with PBS (Thermo Fisher Scientific) harvested by centrifugation, and the supernatant was completely aspirated. A volume of protein removal reagent was added, and the mixture was thoroughly vortexed and measured using the GSH and GSSG assay kit (Beyotime, S0053) according to the manufacturer’s instructions. The cells were collected and lysed for protein extraction, and protein concentrations were measured using the bicinchoninic acid assay (BCA) (Thermo Fisher Scientific). For the MDA (Solarbio, BC0025) and SOD (Solarbio, Beijing, China, BC017S) activity assays, cells were gently resuspended by pipetting and collected into a transferred to centrifuge tubes. After centrifugation at 1000× *g* for 5 min, at 4 °C, the supernatant was discarded. Radio-immunoprecipitation assay (RIPA) lysis buffer (Solarbio, BC0025) (1 mL per 5 × 10^6^ cells) was added to the cell pellet, followed by ultrasonication (3 × 10 s pulses at 20% amplitude) or mechanical disruption. The lysate was centrifuged at 8000× *g* for 10 min at 4 °C and the supernatant was aliquoted and stored on ice for immediate analysis. The NanoDrop 2000c spectrophotometer (Thermo Fisher Scientific) was preheated for >30 min and calibrated to zero using distilled water. Assays were performed according to the manufacturer’s protocol (Solarbio, BC0025 for MDA; BC017S for SOD), with absorbance values normalized to cell counts. MDA content (nmol/10^4^ cells) and SOD enzyme activity (U/10^4^ cells) were calculated based on the protein concentration determined by BCA (Thermo Fisher Scientific).

### 2.5. Detection of Intracellular NO Level

Cells were collected and lysed with homogenization buffer. After centrifugation at 4 °C for 10 min at 12,000× *g*, the supernatant was stored separately to avoid interference from hemolysis or impurities. The nitrate reductase working solution (Solarbio, BC1475) and chromogenic agent were prepared according to the kit (Solarbio, BC1475) protocol. Sample supernatants, gradient standards, and working solution were added sequentially to a 96-well plate. After gentle mixing, the plate was incubated at 37 °C for 30 min. The reaction was terminated with stop solution and absorbance at 550 nm was measured using a Multiskan GO enzyme-linked immunosorbent detector (Thermo Fisher Scientific). NO content (expressed as μ mol/mL) was calculated from the standard curve plotted with optical density (OD) values.

### 2.6. Western Blotting

Following cell homogenization using a sonic dismembrator Gedong XF (Model 200, Gedong XF Biotechnology Company, Wuhan, Hubei, China), the supernatant was collected via high-speed centrifugation. Protein concentration was determined using the BCA (Thermo Fisher Scientific). Subsequently, 50 μg of protein samples were separated by sodium dodecyl sulfate polyacrylamide gel electrophoresis (SDS-PAGE) and transferred onto nitrocellulose membranes (MilliporeSigma, Burlington, MA, USA). The membranes were blocked with non-fat milk and then incubated overnight at 4 °C with anti-PRDX4 (Abclonal, Wuhan, Hubei, China, A1486), anti-PRDX1 (Cell Signaling Technology (CST), #8499), anti-PRDX2 (CST, #46855), and anti-β-actin (Proteintech, Chicago, IL, USA, 81115-1-RR) primary antibodies. Target protein bands were detected using enhanced chemiluminescence (ECL) (Thermo Fisher Scientific).

### 2.7. qPCR Detection of Prdx4 Transcriptional Levels in Cells

To detect the transcriptional level of the Prdx4 gene in cells, total cellular RNA was extracted with a commercial RNA isolation kit (Takara Bio Inc., Shiga, Japan) and verified for purity and integrity, followed by reverse transcription of total RNA into complementary DNA (cDNA) using the PerfectStart Green quantitative real-time PCR (qPCR) SuperMix (TransGen Biotech, Beijing, China, AQ601) according to the manufacturer’s protocol. qPCR was performed with the same kit using β-actin (forward primer: 5′-GCTGTGCTATGTTGCTCTAG-3′, reverse primer: 5′-CGCTCGTTGCCAATAGTG-3′) as the internal reference gene and each sample was analyzed in triplicate with relative Prdx4 transcriptional levels (forward primer: TCCTGTTGCG-177 GACCGAATC, reverse primer: CCACCAGCGTAGAAGTGGC) calculated via the 2^−ΔΔCt^ method and statistical analysis conducted using GraphPad Prism 8.0 software (GraphPad Software, San Diego, CA, USA) [35]. 

### 2.8. PRDX4 Enzyme Activity Assay

In order to evaluate the effect of EB on PRDX4 enzyme activity, pure PRDX4 protein (R&D Systems, Minneapolis, MN, USA) was divided into the control group (DMSO, Sigma-Aldrich) and EB (0, 5, 10, 20, and 50 μM)-treated group. After initial addition of equal amount of the H_2_O_2_ substrate, EB was added to the protein and incubated at 37 °C for 30 min. H_2_O_2_ (Sigma-Aldrich) content was detected at the starting point and the end point, respectively, and the H_2_O_2_ clearance rate was calculated to characterize the enzymatic activity. 

### 2.9. Cycloheximide Chase Assay

RAW264.7 cells were stimulated with 1 μg/mL LPS and pretreated with EB (8 μM) or DMSO for 12 h, followed by CHX treatment (40 μg/mL) for 0 to 24 h to inhibit de novo protein synthesis. Protein samples were collected at 0, 6, 12, and 24 h after the treatment of CHX and the effect of EB on PRDX4 levels was verified using Western blotting. The relative density of the protein bands was quantified.

### 2.10. Synthesis and Bioactivity Study of the EB Fluorescent Probe

The EB-P was synthesized by introducing an alkyne handle at the C-terminus of EB [25]. A total of 111 mg of EB (0.240 mmol) was dissolved in 4 mL of DMF (Sigma-Aldrich). Subsequently, 171 mg of 2-(7-azabenzotriazol-1-yl)-1,1,3,3-tetramethyluronium hexafluorophosphate (HATU) (Sigma-Aldrich, St. Louis, MO, USA) (0.450 mmol) and 0.166 mL of triethylamine (TEA) (Sigma-Aldrich) (1.19 mmol) were added to the solution. The reaction mixture was stirred under dark conditions at room temperature for 12 h. Reaction progress was monitored by TLC using a CH_2_Cl_2_:CH_3_OH (25:1) (Sigma-Aldrich) solvent system until complete consumption of starting material was observed. The reaction mixture was then diluted with approximately 10 mL of saturated ammonium chloride solution (Sigma-Aldrich), resulting in precipitation of a solid product. The crude product was collected by vacuum filtration. Purification was performed via silica gel column chromatography (Haiyang, Qingdao, China) using petroleum ether:ethyl acetate (3:1) (Sigma-Aldrich) as the eluent, yielding compound 3 (94.1 mg, 79.7% yield). The purified compound was stored at 4 °C in the dark for subsequent use.

### 2.11. Labeling and Competition Assay

For the *in vitro* fluorescent labeling and competition assay, activated Raw264.7 cell pellets were collected and lysed using 0.1% triton/PBS (Thermo Fisher Scientific) buffer containing 1× protease inhibitor cocktail (Sigma-Aldrich, cOmplete™ Mini, EDTA-free, 04693159001) (1 tablet/10 mL). The experiment comprised the blank control group, EB-P probe labeling group, and EB pre-treatment competition group. Cells in the competition group were pre-treated with 400 μM EB for 2 h prior to probe addition. Subsequently, each group received different probe concentrations, blank group, 0 μM; labeling group, 50 μM EB-P; and competition group, 50 μM EB-P for another 2 h. The labeling reaction was performed at 37 °C for the designated period. Then the click chemistry reaction mixture (0.1 mM tris (benzyltriazolylmethyl) amine (TBTA) (Sigma-Aldrich), 1 mM CuSO_4_ (Sigma-Aldrich, St. Louis, MO, USA), 50 μM tetramethylrhodamine (TAMRA)-N3 (Sigma-Aldrich), and 1 mM tris(2-carboxyethyl)phosphine (TCEP) (Sigma-Aldrich) was added and incubated at 29 °C for 1 h following by SDS-PAGE and visualization analysis.

### 2.12. Protein Target Identification

To identify the target proteins of EB, we employed a pull-down assay coupled with liquid chromatography tandem mass spectrometry (LC-MS/MS) analysis as previous reported [36,37]. RAW264.7 cells were pre-incubated with EB for 2 h, followed by co-incubation with EB-P for 2 h to establish the EB group and probe labeling group (without EB). Subsequently, a click chemistry reaction mixture (0.1 mM TBTA, 1 mM CuSO_4_, 50 μM biotin-N3 and 1 mM TCEP) was added to the protein solution. After 1 h incubation at 29 °C, proteins were precipitated and resuspended in 0.1% SDS (Bio-Rad, Hercules, CA, USA). Following high-speed centrifugation, the supernatant was incubated overnight at 4 °C with streptavidin magnetic beads (Thermo Fisher Scientific). The bead-bound protein complexes were digested into peptides and subjected to dimethyl labeling. Protein identification was ultimately performed using an Orbitrap Fusion Lumos mass spectrometer (Thermo Fisher Scientific) as previously reported [38]. 

### 2.13. Plasmid Construction and Protein Purification

The mouse-derived Prdx4 gene (GenBank Accession No. NM_001252626) and its mutant cDNA variants were synthesized by Sangon Biotech (Shanghai, China). Protein expression was induced by adding 0.4 mM isopropyl-β-d-thiogalactoside (IPTG) (Sigma-Aldrich). After bacterial cell lysis via ultrasonication, the PRDX4 protein was purified using Ni-iminodiacetic acid (Ni-IDA) affinity chromatography (HisTrap™ FF column, Cytiva, Marlborough, MA, USA). The target protein was eluted with imidazole-containing buffer (50 mM imidazole, 150 mM NaCl, pH 7.4) (Sigma-Aldrich) and collected for SDS-PAGE analysis. Following dialysis, the purified protein was filtered through a 0.22 μm membrane (Millipore, Burlington, MA, USA). The purity of the recombinant protein was confirmed to be greater than 90% by Coomassie brilliant blue (Bio-Rad) staining.

### 2.14. Fluorescence Co-Localization Assay

To visualize the binding of the EB-P probe to the target protein PRDX4, we performed immunofluorescence imaging to investigate the co-localization and interaction between EB and PRDX4. The probe labeling and click chemistry steps were carried out as previously described [38]. Cells were incubated with primary anti-PRDX4 antibody (1:500 dilution) overnight at 4 °C. The next day, samples were washed three times with 1 × TBST (5 min per wash) (Bio-Rad Laboratories, Hercules, CA, USA). Then fluorescent secondary antibody (goat anti-rabbit IgG) (Thermo Fisher Scientific, Catalog No.: 31460) (1:1000 dilution) was applied. Incubation in dark at room temperature for 2 h followed by three additional TBST washes (5 min each). Hoechst 33342 (Thermo Fisher Scientific) (1:1000 dilution) was added for nuclear staining. Finally, samples were mounted using 90% glycerol (Sigma-Aldrich) and imaged with a confocal microscope (Olympus FV3000, Olympus Corporation).

### 2.15. Cellular Thermal Shift Assay (CETSA)

Following established protocols, we performed cellular thermal shift assay (CETSA)-Western blot (CETSA-WB) experiments. RAW264.7 cells were lyzed using cell lysis buffer to extract protein and then each group was aliquoted into many PCR tubes and divided into the control and EB-treated group (400 μM). After incubating the cell lysate with EB for 4 h at 37 °C, samples underwent temperature gradient heating from 37 °C to 82 °C (37, 42, 47, 52, 57, 62, 67, 72, 77, and 82 °C) for 1 h in the PCR thermocycler (Veriti™ Thermal Cycler, Thermo Fisher Scientific). Following heating, lysates were centrifuged at 20,000× *g* for 10 min at 4 °C. Supernatants were collected for Western blot analysis using anti-PRDX4 as the primary detection antibody.

### 2.16. PRDX4-EB Binding Affinity Test by Microscale Thermophoresis 

To quantify PRDX4-EB binding affinity via microscale thermophoresis (MST) (NanoTemper Monolith NT.115, NanoTemper Technologies, Munich, Germany). His-tagged PRDX4 was nitrilotriacetic acid labeled with nitrilotriacetic acid (NTA) (Sigma-Aldrich) and diluted to 50 nM. EB was 10-fold serially diluted (0.01–1000 μM, ≤5% DMSO) to span ~2 orders of magnitude around the predicted dissociation constant (Kd). 10 μL EB dilutions were mixed with 10 μL 50 nM labeled PRDX4 (25 °C, 5 min equilibrium), centrifuged (17,000× *g*, 5 min), and supernatants loaded into MST capillaries (no air bubbles). Using an MST instrument, the laser power and MST intensity were optimized and a pre-scan was performed to confirm uniform fluorescence before initiating the measurement. Upon exposure to the IR laser, the instrument automatically records the time-dependent changes in fluorescence signal during the localized heating and subsequent cooling phases. For data analysis, the acquired signals were normalized $F_{norm}=\frac{F_{hot}}{F_{cold}}$ and subjected to nonlinear fitting (e.g., using the Hill model or Kd model) to calculate key parameters such as the Kd, Hill coefficient, and stoichiometry, thereby quantitatively characterizing the affinity and specificity of the molecular interaction.

### 2.17. RNA Interference Experiment

PrdX4 siRNA (sense strand, GAAAGCAGAUAG) was synthesized by Sangon Biotech. siRNA and the corresponding control were diluted in serum-free DMEM medium (Thermo Fisher Scientific, 10829023) and transfected into RAW264.7 cells using Lipofectamine 2000 (Thermo Fisher Scientific) according to the manufacturer’s instructions. After 48 h of incubation, cells were treated with 1 μg/mL LPS and EB as required by the experimental design. Finally, cells were harvested for Western blot analysis and culture supernatants were collected for measurement of inflammatory cytokine levels.

### 2.18. Binding Site Identification

To determine the direct interaction sites between EB and the PRDX4 protein (Abcam, Cambridge, UK), 100 μg of purified PRDX4 protein was incubated with 2 mM EB for 2 h. Subsequently, 5 mM DTT (Sigma-Aldrich) was added to reduce disulfide bonds, followed by the addition of 20 mM iodoacetamide (IAA) (Sigma-Aldrich) after 30 min to perform alkylation reactions. To remove drug and reagent interference, a mixture of 300 μL methanol (Sigma-Aldrich), 100 μL chloroform (Sigma-Aldrich), and 300 μL double-distilled water (ddH_2_O) was added. The solution was left undisturbed to precipitate the PRDX4 protein. Finally, the precipitated protein was digested with 1 μg of trypsin (Thermo Fisher Scientific, V511A) into peptide fragments. The peptides were desalted using a C18 column (Waters, Milford, MA, USA) and subsequently analyzed by MS.

### 2.19. Molecular Docking

Crystal structure of PRDX4 Protein Data Bank (PDB ID, 3VWU) was obtained from the Research Collaboratory for Structural Bioinformatics (RCSB) Protein Data Bank. Schrödinger Glide was employed to simulate the binding modes between EB and PRDX4 [39]. Subsequently, the Epik and OPLS4 force field modules were employed to predict protonation states [40], preprocess the protein under pH 7.4, optimize hydrogen bond networks, and perform energy minimization. The three-dimensional structures were constructed using Pymol and the Maestro LigPrep module with the OPLS4 force field at pH 7.4.

### 2.20. Statistical Analysis

Statistical analysis of data was performed by Student’s *t* test and one-way analysis of variance (ANOVA) followed by Tukey’s post hoc test. All experiments were performed for at least three independent biological replicates. Statistical analyses was conducted using GraphPad Prism 9.0. Data are represented as the means ± standard deviation (SD). * *p* < 0.05; ** *p* < 0.01; *** *p* < 0.001; or **** *p* < 0.0001 was significant. ns, not significant.

## 3. Results

### 3.1. EB Effectively Alleviates Oxidative Stress in LPS-Stimulated RAW264.7 Cells

**Figure 1:**
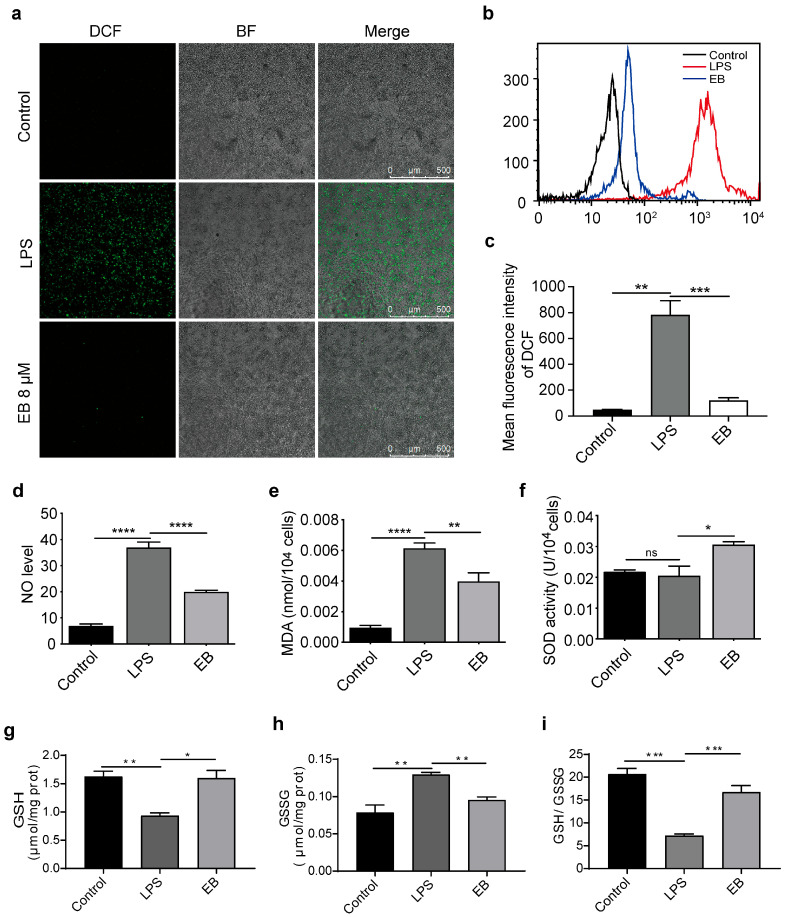
Effect of EB on the antioxidant capacity of LPS-induced RAW264.7 cells. (**a**) LPS induces ROS accumulation in RAW264.7 cells, which is reversed by EB treatment for 24 h. (**b**,**c**) DCF fluorescence intensities of ROS in LPS-induced RAW264.7 cells quantified by flow cytometry. (**d**,**e**) The effect of EB intervention on LPS-induced levels of NO (**d**) and MDA (**e**) in RAW264.7 cells. (**f**) Effect of EB intervention on SOD activity in LPS-induced RAW264.7 cells. (**g**) GSH and (**h**) GSSG content. (**i**) GSH/GSSG ratios. All error bars, mean values ± SD. * *p* < 0.05, ** *p* < 0.01, *** *p* < 0.001, **** *p* < 0.0001; ns, no significance.

To evaluate the antioxidant efficacy of EB, we established an LPS-induced oxidative stress model in RAW264.7 macrophages and measured key oxidative stress parameters including intracellular oxidants (ROS, NO, and RNS), oxidative damage markers MDA and core antioxidants (enzymatic SOD and non-enzymatic GSH, together with its oxidized form GSSG). As illustrated in Figure 1a, LPS stimulation markedly increased the population of DCF fluorescent-positive cells, indicating substantial ROS accumulation. This effect was substantially reversed upon treatment with 8 μM EB. Quantitative analysis of the DCF fluorescence signal by flow cytometry confirmed that EB significantly attenuated the LPS-induced ROS elevation (Figure 1b,c). Consistently, LPS stimulation also triggered a dramatic increase in NO production (Figure 1d), a key pro-oxidant that synergizes with ROS to exacerbate oxidative damage [41]. Notably, EB treatment significantly reduced NO levels. Furthermore, EB treatment resulted in a distinct reduction in MDA content, a marker of lipid peroxidation, compared to the LPS-treated model group (Figure 1e). Conversely, the activity of SOD, a pivotal antioxidant enzyme, was markedly elevated in EB-treated cells (Figure 1f) [42]. In addition to SOD activity, we further detected GSH (Figure 1g) and GSSG (Figure 1h) levels to comprehensively evaluate the antioxidant network. EB treatment significantly increased the GSH/GSSG ratio (Figure 1i) compared to the LPS group, indicating that EB enhances the cellular antioxidant defense system.

### 3.2. Identification of EB Target Proteins Using Activity-Based Protein Profiling

**Figure 2:**
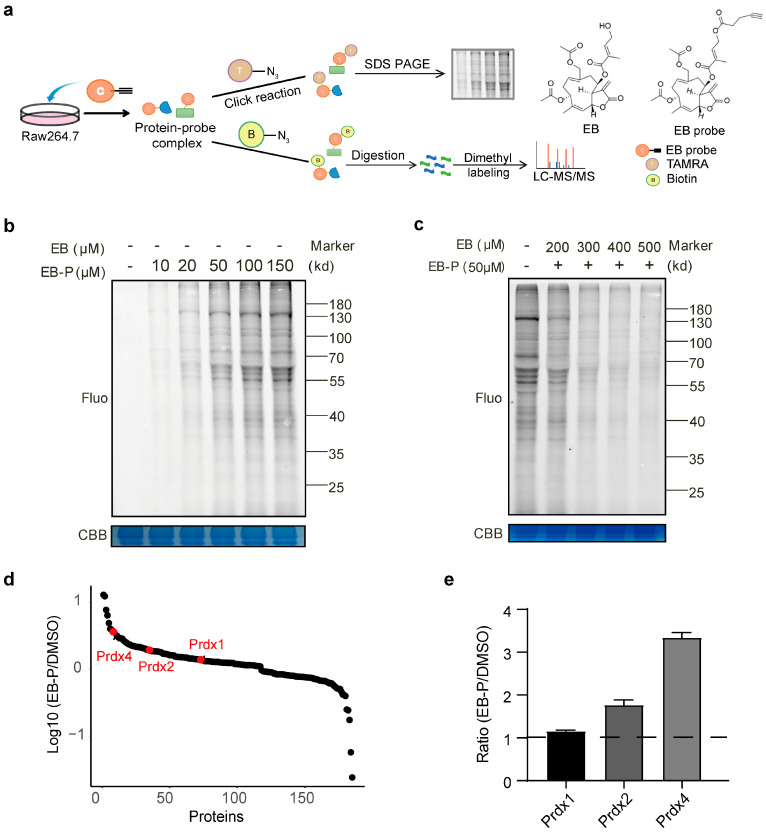
Identification of EB target proteins in RAW264.7 cells. (**a**) Schematic workflow for identifying EB target proteins. (**b**) Target proteins labeling efficiency with EB-P at different concentrations. (**c**) Target proteins labeled with 50 µM EB-P and competitively inhibited by ten times of concentrations of EB. (**d**) Proteins identified in LPS-induced RAW264.7 cells. (**e**) Relative abundance ratios of PRDX family proteins as determined by ABPP. All error bars, mean values ± SD.

To elucidate the molecular targets underlying the antioxidant activity of EB, we employed ABPP to identify its direct binding partners in RAW264.7 cells. An active EB probe (EB-P) was synthesized by incorporating a clickable alkyne handle, preserving the core structure for target engagement (Figure 2a). This design enabled subsequent conjugation to TAMRA-N3 via click chemistry for in-gel fluorescence detection as well as to biotin-azide for affinity enrichment and identification of bound proteins by LC-MS/MS (Figure 2a). Initial optimization of labeling conditions in RAW264.7 cell lysates revealed efficient target engagement at an EB-P concentration of 50 μM (Figure 2b). Competitive profiling experiments demonstrated that pre-incubation with increasing concentrations of native EB dose-dependently reduced the fluorescence intensity of EB-P-labeled proteins, confirming specific binding (Figure 2c). Based on these results, 50 μM EB-P and 400 μM EB were selected as optimal concentrations for subsequent labeling and competition experiments, respectively. Following affinity enrichment and LC-MS/MS analysis, candidate target proteins were filtered based on a significant reduction in the competition group (fold change < 0.8, *p* value < 0.05). Among the enriched proteins, three isoforms of the peroxiredoxin (PRDX) family PRDX1, 2, and 4 were notably identified as redox-related targets (Figure 2d). Importantly, PRDX4 exhibited the highest probe-to-competition ratio among all identified proteins, highlighting it as the most prominently engaged target by EB under our experimental conditions (Figure 2e).

### 3.3. EB Directly Binds to PRDX4

**Figure 3:**
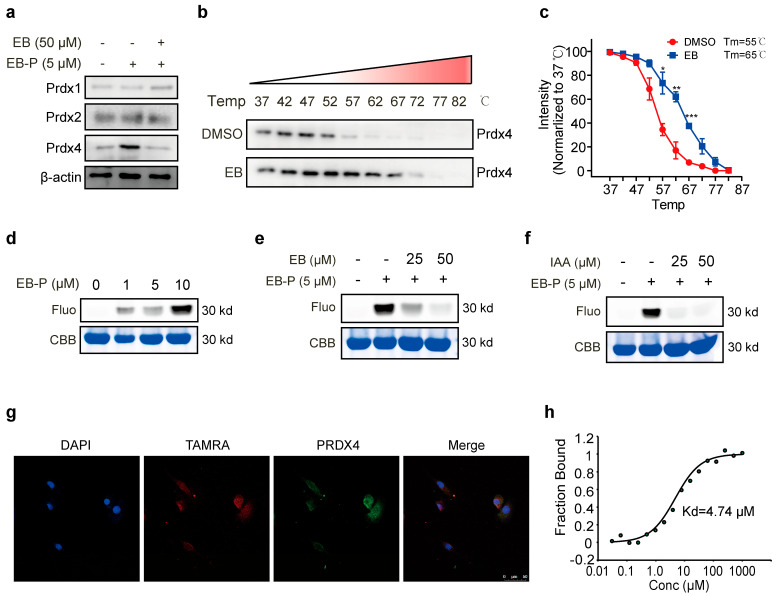
Direct interaction between EB and PRDX4. (**a**) Pull-down-WB assay showing enrichment of PRDX1, 2, and 4 by EB-P in Raw264.7 cells with or without competition by EB. (**b**) The CETSA-WB assay. (**c**) Thermal shift curves of PRDX4 with increased thermal stability by immunoblotting in cell lysates. Blue curve represents EB treatment and the red curve represents the DMSO treatment control. (**d**) In vitro labeling of PRDX4 by EB-P. (**e**) Competitive inhibition of EB-P labeling to PRDX4 in the presence or absence of EB. (**f**) Competition assay demonstrating EB-P labeling of PRDX4 with or without IAA. (**g**) Co-localization of EB-P (Red) and PRDX4 (green) by immunofluorescence staining using the PRDX4 antibody. (**h**) MST measurement of the binding affinity between EB and PRDX4. All error bars, mean values ± SD. * *p* < 0.05, ** *p* < 0.01, *** *p* < 0.001; ns, no significance.

To biochemically validate the interaction between EB and PRDX4, we performed a pull-down assay combined with Western blot analysis. The results demonstrated that PRDX4 was efficiently enriched by the EB-P probe and this enrichment was substantially diminished in the presence of excess native EB, confirming the specificity of the EB-PRDX4 interaction (Figure 3a). Furthermore, CETSA revealed that EB treatment significantly increased the thermal stability of PRDX4 (Figure 3b,c), providing independent evidence of direct binding within the cellular milieu. We next sought to recapitulate this interaction in a purified system. Incubation of recombinant PRDX4 protein with EB-P resulted in a concentration-dependent labeling with robust signal observed at 5 μM EB-P (Figure 3d). This labeling was effectively competed away by pre-incubation with an excess of native EB (Figure 3e). Notably, pre-treatment with the cysteine-alkylating agent IAA also abolished EB-P labeling (Figure 3f), strongly suggesting that EB binds covalently to cysteine residues on PRDX4. Finally, to visualize this interaction in intact cells, we performed fluorescence co-localization studies. Confocal microscopy revealed a significant overlap between the signal from EB-P (red) and immunofluorescently labeled PRDX4 (green) (Figure 3g). This spatial co-localization provides further corroborating evidence for the direct interaction between EB and PRDX4 in a physiological cellular context. Further more, MST was performed to determine the direct binding affinity between EB and PRDX4. The results showed a Kd of 4.74 μM (Figure 3h), demonstrating that EB and PRDX4 bind directly with high affinity.

### 3.4. EB Covalently Modifies Cys54 and Cys248 Residues of PRDX4

**Figure 4:**
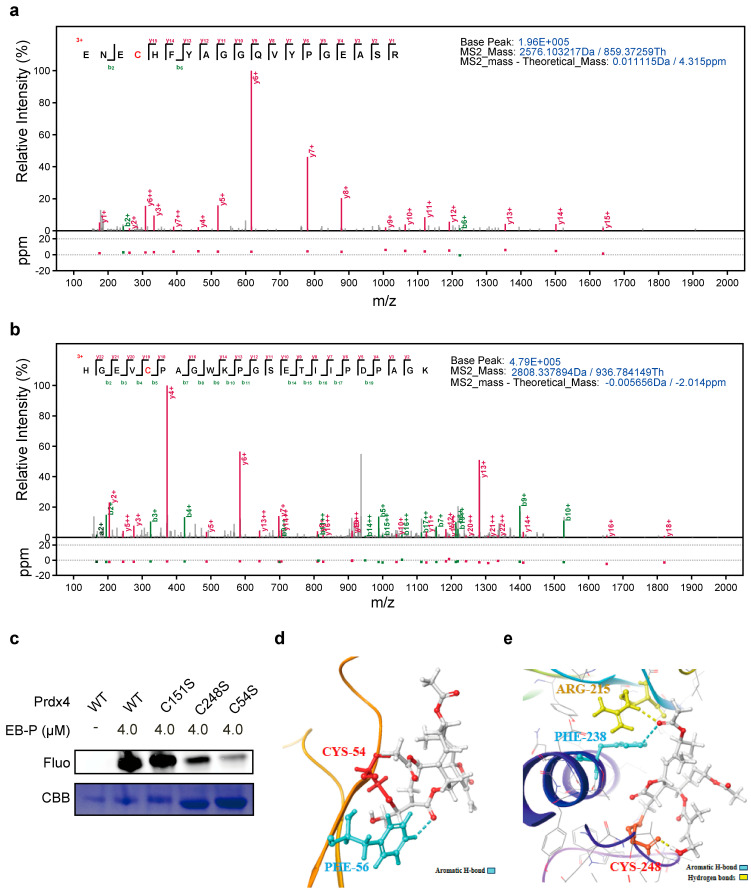
Covalent binding of EB to Cys54 and Cys248 residues of PRDX4. (**a**,**b**) MS/MS spectra of precursor ions used for precise sequencing and localization of EB binding sites. (**c**) Labeling of PRDX4 and its mutants (PRDX4-C54S, PRDX4-C151S, and PRDX4-C248S) using the EB-P probe. (**d**,**e**) Binding site interactions in the PRDX4/EB complex generated by molecular docking.

To precisely map the binding sites of EB on PRDX4, recombinant PRDX4 protein was incubated with EB and subjected to tryptic digestion followed by LC-MS/MS analysis. This approach identified two specific peptides with molecular weights of 2576.1 and 2808.3 Da, which exhibited covalent modification by EB (Figure 4a,b). These peptides were unambiguously assigned to sequences containing the catalytic cysteine residues Cys248, Cys54, and Cys151, respectively. Notably, no other cysteine-containing peptides showed EB modification, indicating a spatially selective targeting of these key residues. To further validate the functional importance of these cysteines, we generated a series of PRDX4 point mutants (C54S, C151S, and C248S) and performed labeling experiments with the EB-P probe. The results demonstrated a substantial loss of EB-P binding to both the PRDX4-C54S and PRDX4-C248S mutants, while binding to the C151S mutant remained comparable to the wild-type protein (Figure 4c). This confirms that Cys54 and Cys248 are the primary sites of covalent EB modification. Subsequently, molecular docking simulations were performed to elucidate the binding mode of EB within the catalytic pockets of PRDX4 (PDB: 3VWU). As shown in Figure 4d,e, EB was favorably accommodated in both the Cys54 and Cys248 binding sites. The α,β-unsaturated carbonyl moiety of EB formed a covalent bond with the thiol group of each cysteine via a Michael addition reaction. The binding pose was further stabilized by specific non-covalent interactions, including hydrogen bonding with ARG215 and aromatic interactions with PHE238 and PHE5. Collectively, these integrated structural and biochemical analyses establish that EB forms specific covalent adducts with the catalytic cysteines Cys54 (Glide score-1.914) and Cys248 (Glide score-2.516) of PRDX4, thereby providing a molecular basis for its antioxidant mechanism [43].

### 3.5. EB Exerts Antioxidant Effects by Binding and Stabilizing PRDX4

**Figure 5:**
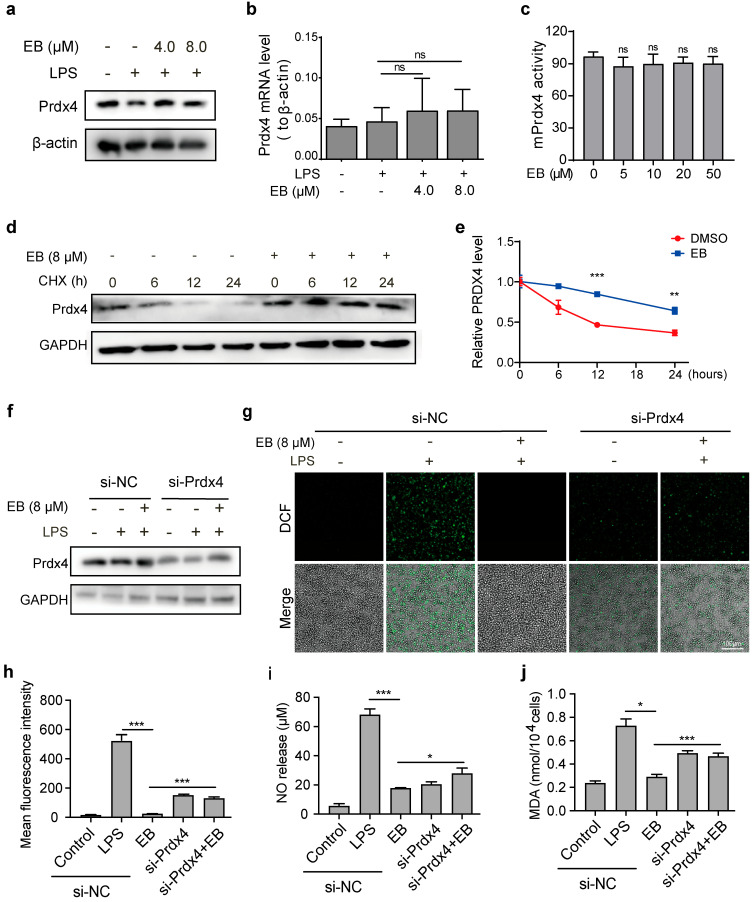
PRDX4 knockdown attenuates the antioxidant effects of EB. (**a**) PRDX4 protein levels in RAW264.7 cells detected by Western blotting after LPS stimulation for 24 h with EB treatment. (**b**) Prdx4 mRNA levels in cells. (**c**) Peroxidase activity of PRDX4 (150 μg/mL) with EB (0–50 μM) in H_2_O_2_ reduction assay. (**d**) Representative Western blot images showing the dynamic degradation of PRDX4 protein in LPS-stimulated Raw264.7 cells after treatment with CHX (40 μg/mL) for the indicated time points (0, 6, 12, and 24 h) without or with EB pre-treatment. (**e**) Quantitative analysis of PRDX4 protein levels. (**f**) Western blot analysis of PRDX4 protein levels in RAW264.7 cells transfected with si-NC or si-PRDX4. (**g**) ROS levels in LPS-induced RAW264.7 cells detected by immunofluorescence staining after EB treatment. (**h**) Quantification of DCF fluorescence intensity in RAW264.7 cells treated with EB and LPS. (**i**,**j**) Levels of NO in cell culture supernatants (**i**) and intracellular MDA (**j**). All error bars, mean values ± SD, * *p* < 0.05, ** *p* < 0.01, *** *p* < 0.001; ns, no significance.

To elucidate the functional role of PRDX4 in EB-mediated antioxidant activity, we first examined the effect of EB on PRDX4 protein expression. Western blot analysis demonstrated that EB treatment at 4 and 8 μM significantly upregulated PRDX4 protein levels in LPS-stimulated RAW264.7 cells (Figure 5a). To verify whether EB affects the transcriptional level of PRDX4, we performed qPCR experiments. The results showed that EB had no impact on PRDX4 transcription (Figure 5b). We also conducted in vitro enzyme activity assays using the purified PRDX4 protein and the results revealed no significant difference in the enzyme activity rate between the PRDX4 plus EB group and the PRDX4-only group (Figure 5c). These results confirm that EB does not directly alter the intrinsic enzymatic activity of PRDX4 (Figure 5c) or its transcriptional level (Figure 5b). To further verify whether EB enhances PRDX4 post-translational stability, we performed a CHX-chase assay. Western blot analysis showed that EB treatment significantly slowed the degradation rate of PRDX4 (Figure 5d,e). These results confirm that the enhanced total antioxidant activity of PRDX4 is attributed to EB-mediated improvement of PRDX4 post-translational stability. To determine whether PRDX4 is functionally required for the antioxidant effect of EB, we employed siRNA-mediated knockdown. Transfection with specific siRNA effectively reduced PRDX4 protein expression compared to the negative control (si-NC) (Figure 5f). Under these conditions of PRDX4 depletion, we re-evaluated key oxidative stress parameters. As shown in Figure 5g,h, PRDX4 knockdown significantly attenuated the capacity of EB to suppress LPS-induced ROS production. Furthermore, the suppressive effects of EB on LPS-induced NO and MDA production were also significantly reversed upon PRDX4 knockdown (Figure 5i,j). The attenuation of the antioxidant efficacy of EB across multiple independent readouts following PRDX4 depletion underscores the essential role of this protein in mediating the cellular response to EB. In summary, these results demonstrate that EB binds to PRDX4, enhances its protein stability and expression, and subsequently augments the cellular antioxidant capacity. The loss of the protective effects of EB upon PRDX4 knockdown provides direct genetic evidence that PRDX4 is a critical functional target through which EB alleviates oxidative stress.

## 4. Discussion

EB, a principal bioactive sesquiterpene lactone contains an α, β-unsaturated ketone structure and exerts biological functions by covalently binding to the active sites of various proteins to exert diverse pharmacological properties [44,45,46,47]. Our study demonstrates that EB significantly mitigates oxidative stress in LPS-stimulated RAW264.7 macrophages. We observed reduced levels of the oxidative damage markers ROS, NO, and MDA coupled with enhanced enzymatic antioxidant SOD activity and restored GSH/GSSG balance representing the core non-enzymatic redox buffering system.

We identified PRDX4 as a primary target of EB in macrophages. PRDX4, a key member of the peroxiredoxin family, functions as a critical intracellular antioxidant enzyme by scavenging peroxides (e.g., H_2_O_2_ and lipid peroxides) and maintaining cellular redox homeostasis [48]. The dynamic equilibrium between ROS production and antioxidant capacity is crucial for cellular function and its disruption is a hallmark of numerous chronic diseases [49,50]. Research has shown that PRDX4 plays important roles in various diseases by inhibiting oxidative stress and reducing levels of inflammatory cytokines such as TNF-α and IL-1β, thereby suppressing disease progression [51,52,53,54,55]. 

Our mechanistic investigation further delineated the molecular basis of EB-PRDX4 interaction. Through a combination of MS analysis and site-directed mutagenesis, we identified Cys54 and Cys248 as the specific cysteine residues covalently modified by EB. This modification pattern is generally considered to potentially affect protein function [56,57]; however, we observed enhanced antioxidant and anti-inflammatory activities of PRDX4 in the present study. To directly validate the effect of EB on PRDX4 stability, we performed a CHX-chase assay. These experiments confirmed that EB treatment significantly prolonged the half-life of PRDX4 protein. Our further results confirmed that EB treatment exerted no significant effects on PRDX4 activity and transcription level but concurrently increased PRDX4 protein levels. Covalent binding, a common interaction mode between natural products and target proteins, not only inhibits enzyme activity but also alters the spatial conformation of PRDX4 [38]. This conformational change modulates PRDX4 interactions with intracellular molecules involved in protein degradation, thereby reducing degradation and ultimately leading to the accumulation of PRDX4 protein levels and enhanced antioxidant capacity.

Sesquiterpene lactones, as a highly active family of natural products, exert their redox-regulating functions mainly through targeting sulfhydryl (SH) groups and modulating ROS homeostasis [58,59,60]. However, they only indirectly affect redox balance and no direct targeted post-translational modification regulation of the peroxidase family has been identified. Among the reported specific modulators of PRDX4, the mechanisms of action mainly fall into regulating protein stability and degradation [61] and modulating post-translational modifications to inhibit PRDX4 oligomerization, thereby blocking its interaction with downstream targets [62]. This study is the first to report that sesquiterpene lactones directly target and bind to PRDX4 to regulate its protein stability and enhance antioxidant capacity.

Although our work provides comprehensive evidence that PRDX4 is a direct functional target of EB, it still has certain limitations. While our CHX assays confirm EB prolongs PRDX4 half-life, the specific molecular mechanisms such as whether the ubiquitin-proteasome pathway is involved remain to be further verified for example via co-immunoprecipitation of PRDX4 with ubiquitin or proteasomal subunits. In addition, the physiological relevance of the identified binding sites as well as the therapeutic efficacy of EB need to be validated in more complex physiological contexts, particularly using PRDX4 knockout animal models. Furthermore, future work should complement these data with subtype-specific ROS detection (e.g., MitoSOX for mitochondrial superoxide) and additional lipid peroxidation markers (e.g., 4-hydroxynonenal (4-HNE)) to strengthen the specificity of our oxidative damage conclusions. Future studies should also explore the potential off-target effects of EB and its broader impacts on the cellular redox network.

In conclusion, we identified PRDX4 as a direct target protein of EB in macrophages for the first time. EB binds covalently to the cysteine 54 and cysteine 248 sites of PRDX4, prolonging PRDX4 protein half-life and enhancing both enzymatic and non-enzymatic antioxidant capacity—expanding the scope of covalent targets for natural product-based redox modulators and laying a new molecular foundation for PRDX4-targeted drug development. Based on this study, in the future, we can verify the efficacy of EB targeting PRDX4 in models of ischemia-reperfusion injury, neurodegenerative and inflammatory diseases, and study the synergistic effects between drugs targeting PRDX4 and existing redox drugs to promote the development of combined therapies.

## Data Availability

The original contributions presented in this study are included in the article. Further inquiries can be directed to the corresponding author.

## References

[1] Duni A., Liakopoulos V., Roumeliotis S., Peschos D., Dounousi E. (2019). Oxidative Stress in the Pathogenesis and Evolution of Chronic Kidney Disease: Untangling Ariadne’s Thread. Int. J. Mol. Sci..

[2] Niranjan R. (2014). The role of inflammatory and oxidative stress mechanisms in the pathogenesis of Parkinson’s disease: Focus on astrocytes. Mol. Neurobiol..

[3] Chen S., Li L., Peng C., Bian C., Ocak P.E., Zhang J.H., Yang Y., Zhou D., Chen G., Luo Y. (2022). Targeting Oxidative Stress and Inflammatory Response for Blood-Brain Barrier Protection in Intracerebral Hemorrhage. Antioxid. Redox Signal..

[4] Luo M., Zhao F.K., Wang Y.M., Luo Y. (2024). Nanomotors as Therapeutic Agents: Advancing Treatment Strategies for Inflammation-Related Diseases. Chem. Rec..

[5] Wang X., Shi H., Li X., Feng Y. (2025). Macrophages in rosacea: Pathogenesis and therapeutic potential. Front. Immunol..

[6] Lee H.J., Ji K.Y., Jung D.H., Lee J.Y., Choi H., Kim Y., Lee W., Kim T., Chae S., Hong S.W. (2025). Immunomodulatory Properties and Ameliorative Effects of Pediococcus inopinatus in an Animal Model of Inflammatory Bowel Disease. J. Microbiol. Biotechnol..

[7] Seong H.K., Kim M.J., Fauziah A.N., Jeong H.S., Kim H.J., Yang C.Y., Park S.J., Bae S.Y., Jung S.K. (2025). The Inhibitory Effects of Cordyceps militaris ARA301 Extract on Lipopolysaccharide-Induced Lung Injury in vivo. J. Microbiol. Biotechnol..

[8] Oh Y.C., Jeong Y.H., Ha J.H., Cho W.K., Ma J.Y. (2014). Oryeongsan inhibits LPS-induced production of inflammatory mediators via blockade of the NF-kappaB, MAPK pathways and leads to HO-1 induction in macrophage cells. BMC Complement. Altern. Med..

[9] Jia Y., Zhang Z., Zhang S., Ma X., Ruan Y., Ma B., Wang L. (2024). Effects of polysaccharide from hot-compressed steamed Rehmannia glutinosa on the immune system and gut microbiota in an immunosuppressed mice model. Int. Immunopharmacol..

[10] Murray P.J., Allen J.E., Biswas S.K., Fisher E.A., Gilroy D.W., Goerdt S., Gordon S., Hamilton J.A., Ivashkiv L.B., Lawrence T. (2014). Macrophage activation and polarization: Nomenclature and experimental guidelines. Immunity.

[11] Pacher P., Beckman J.S., Liaudet L. (2007). Nitric oxide and peroxynitrite in health and disease. Physiol. Rev..

[12] Pizzino G., Irrera N., Cucinotta M., Pallio G., Mannino F., Arcoraci V., Squadrito F., Altavilla D., Bitto A. (2017). Oxidative Stress: Harms and Benefits for Human Health. Oxidative Med. Cell. Longev..

[13] Valko M., Leibfritz D., Moncol J., Cronin M.T., Mazur M., Telser J. (2007). Free radicals and antioxidants in normal physiological functions and human disease. Int. J. Biochem. Cell Biol..

[14] Foyer C.H., Noctor G. (2005). Redox homeostasis and antioxidant signaling: A metabolic interface between stress perception and physiological responses. Plant Cell.

[15] Islam M.N., Rauf A., Fahad F.I., Emran T.B., Mitra S., Olatunde A., Shariati M.A., Rebezov M., Rengasamy K.R.R., Mubarak M.S. (2022). Superoxide dismutase: An updated review on its health benefits and industrial applications. Crit. Rev. Food Sci. Nutr..

[16] Zhao H., Zhang R., Yan X., Fan K. (2021). Superoxide dismutase nanozymes: An emerging star for anti-oxidation. J. Mater. Chem. B.

[17] Poole L.B., Hall A., Nelson K.J. (2011). Overview of peroxiredoxins in oxidant defense and redox regulation. Curr. Protoc. Toxicol..

[18] Fujii J., Ikeda Y., Kurahashi T., Homma T. (2015). Physiological and pathological views of peroxiredoxin 4. Free Radic. Biol. Med..

[19] Lee Y.J. (2020). Knockout Mouse Models for Peroxiredoxins. Antioxidants.

[20] Tavender T.J., Springate J.J., Bulleid N.J. (2010). Recycling of peroxiredoxin IV provides a novel pathway for disulphide formation in the endoplasmic reticulum. EMBO J..

[21] Huang Y., Zhang Y., Liu Y., Jin Y., Yang H. (2025). PRDX4 mitigates diabetic retinopathy by inhibiting reactive gliosis, apoptosis, ER stress, oxidative stress, and mitochondrial dysfunction in Müller cells. J. Biol. Chem..

[22] Aihaiti Y., Tuerhong X., Zheng H., Cai Y., Yang M., Xu P. (2022). Peroxiredoxin 4 regulates tumor-cell-like characteristics of fibroblast-like synoviocytes in rheumatoid arthritis through PI3k/Akt signaling pathway. Clin. Immunol..

[23] Jia W., Chen P., Cheng Y. (2019). PRDX4 and Its Roles in Various Cancers. Technol. Cancer Res. Treat..

[24] Matkowski A., Jamiołkowska-Kozlowska W., Nawrot I. (2013). Chinese medicinal herbs as source of antioxidant compounds—Where tradition meets the future. Curr. Med. Chem..

[25] Zou Y., Wang S., Zhang H., Gu Y., Chen H., Huang Z., Yang F., Li W., Chen C., Men L. (2024). The triangular relationship between traditional Chinese medicines, intestinal flora, and colorectal cancer. Med. Res. Rev..

[26] Shao X., Chen Y., Zhang J., Zhang X., Dai Y., Peng X., Fan X. (2025). Advancing network pharmacology with artificial intelligence: The next paradigm in traditional Chinese medicine. Chin. J. Nat. Med..

[27] Fan Z., Du H., Zhou X., Wang C., Zhang M., Sun T., Wang Y., Wang P. (2025). Network Toxicology and Molecular Docking Reveal the Toxicological Mechanisms of DEHP in Bone Diseases. Int. J. Mol. Sci..

[28] Qin W., Yang F., Wang C. (2020). Chemoproteomic profiling of protein-metabolite interactions. Curr. Opin. Chem. Biol..

[29] Wang H., Liu L., Zhang Z., Li C., Wang K., Gao J., Hu Q., Wang W., Li H. (2025). Insights of affinity-based probes for target identification in drug discovery. Eur. J. Med. Chem..

[30] Zhang X.W., Feng N., Liu Y.C., Guo Q., Wang J.K., Bai Y.Z., Ye X.M., Yang Z., Yang H., Liu Y. (2022). Neuroinflammation inhibition by small-molecule targeting USP7 noncatalytic domain for neurodegenerative disease therapy. Sci. Adv..

[31] Yang L., Chen H., Hu Q., Liu L., Yuan Y., Zhang C., Tang J., Shen X. (2022). Eupalinolide B attenuates lipopolysaccharide-induced acute lung injury through inhibition of NF-κB and MAPKs signaling by targeting TAK1 protein. Int. Immunopharmacol..

[32] Bai Q., Wang C., Ding N., Wang Z., Liu R., Li L., Piao H., Song Y., Yan G. (2025). Eupalinolide B targets DEK and PANoptosis through E3 ubiquitin ligases RNF149 and RNF170 to negatively regulate asthma. Phytomedicine.

[33] Khadaroo R.G., Kapus A., Powers K.A., Cybulsky M.I., Marshall J.C., Rotstein O.D. (2003). Oxidative Stress Reprograms Lipopolysaccharide Signaling via Src Kinase-dependent Pathway in RAW 264.7 Macrophage Cell Line. J. Biol. Chem..

[34] Yu H., Jin Y., Jeon H., Kim L., Heo K.-S. (2024). Protective effect of 6′-Sialyllactose on LPS-induced macrophage inflammation via regulating Nrf2-mediated oxidative stress and inflammatory signaling pathways. Korean J. Physiol. Pharmacol..

[35] Chen Y., Chen N., Wang J., Li S. (2023). Effects of Baimuxinol on the inflammation and oxidative stress of LPS-induced RAW264.7 macrophages via regulating the NF-κB/IκBα and Nrf2/ARE signaling pathway. Acta Biochim. Pol..

[36] Livak K.J., Schmittgen T.D. (2001). Analysis of relative gene expression data using real-time quantitative PCR and the 2^−ΔΔCT^ method. Methods.

[37] Huang L., Li G., Zhang Y., Zhuge R., Qin S., Qian J., Chen R., Wong Y.K., Tang H., Wang P. (2024). Small-molecule targeting BCAT1-mediated BCAA metabolism inhibits the activation of SHOC2-RAS-ERK to induce apoptosis of Triple-negative breast cancer cells. J. Adv. Res..

[38] Kuang W., Zhuge R., Song P., Yi L., Zhang S., Zhang Y., Wong Y.K., Chen R., Zhang J., Wang Y. (2025). Eupalinolide B inhibits periodontitis development by targeting ubiquitin conjugating enzyme UBE2D3. MedComm.

[39] Rouen K.C., Narang K., Han Y., Wang D., Jang E., Brunkow S., Yarov-Yarovoy V., MacKerell A.D., Vorobyov I. (2025). Arrhythmia risk predictions from molecular simulations of cardiac ion channel-drug interactions. Biophys. J..

[40] Johnston R.C., Yao K., Kaplan Z., Chelliah M., Leswing K., Seekins S., Watts S., Calkins D., Elk J.C., Jerome S.V. (2023). Epik: p*K*_a_ and Protonation State Prediction through Machine Learning. J. Chem. Theory Comput..

[41] Lijia Z., Zhao S., Wang X., Wu C., Yang J. (2012). A self-propelling cycle mediated by reactive oxide species and nitric oxide exists in LPS-activated microglia. Neurochem. Int..

[42] Ray P.D., Huang B.-W., Tsuji Y. (2012). Reactive oxygen species (ROS) homeostasis and redox regulation in cellular signaling. Cell. Signal..

[43] Fan T., Zhao L., Ma X., Tang J., Duan Q., Zhu X., Liu Y., Jiang J., Li Y., Song D. (2025). Evolution of 8-esterified cycloberberines as a novel class of antibacterial agents against MDR gram-positive strains by targeting DNA polymerase IIIC. Bioorg. Chem..

[44] Huang L., Xu D.Q., Chen Y.Y., Fu R.J., Yue S.J., Yin J.F., Tang Y.P. (2021). Qualitative and quantitative analysis of chemical components in Eupatorium lindleyanum DC. by ultra-performance liquid chromatography-mass spectrometry integrated with anti-inflammatory activity research. J. Sep. Sci..

[45] Lu J., Zheng C., Xue S., Gao Y., Chen G., Shan C., Ding N., Peng G., Li C., Zheng Y. (2024). Comprehensive Comparison of Three Different Medicinal Parts of Eupatorium lindleyanum DC. Using the RRLC-Q-TOF-MS-Based Metabolic Profile and In Vitro Anti-Inflammatory Activity. Molecules.

[46] Li Y., Liu X., Li L., Zhang T., Gao Y., Zeng K., Wang Q. (2023). Characterization of the metabolism of eupalinolide A and B by carboxylesterase and cytochrome P450 in human liver microsomes. Front. Pharmacol..

[47] Zhang Y., Zhang H., Mu J., Han M., Cao Z., Dong F., Wang T., Pan L., Luo W., Li J. (2022). Eupalinolide B inhibits hepatic carcinoma by inducing ferroptosis and ROS-ER-JNK pathway. Acta Biochim. Biophys. Sin..

[48] Drago L., Ferro D., Bakiu R., Ballarin L., Santovito G. (2021). Typical 2-Cys Peroxiredoxins as a Defense Mechanism against Metal-Induced Oxidative Stress in the Solitary Ascidian Ciona robusta. Antioxidants.

[49] Malcangi G., Patano A., Ciocia A.M., Netti A., Viapiano F., Palumbo I., Trilli I., Guglielmo M., Inchingolo A.D., Dipalma G. (2023). Benefits of Natural Antioxidants on Oral Health. Antioxidants.

[50] Jiménez-Peñuela J., Ferraguti M., Martínez-De La Puente J., Soriguer R.C., Figuerola J. (2023). Oxidative status in relation to blood parasite infections in house sparrows living along an urbanization gradient. Environ. Pollut..

[51] Zou X., Liang X., Dai W., Zhu T., Wang C., Zhou Y., Qian Y., Yan Z., Gao C., Gao L. (2024). Peroxiredoxin 4 deficiency induces accelerated ovarian aging through destroyed proteostasis in granulosa cells. Biochim. Biophys. Acta Mol. Basis Dis..

[52] Xuelian L., Panfeng S., Xingxing Z., Lilong M. (2025). Research progress on the role and molecular mechanism of PRDX4 in malignant tumors. Bull. Cancer.

[53] Kocatürk B. (2023). In silico analysis reveals PRDX4 as a prognostic and oncogenic marker in renal papillary cell carcinoma. Gene.

[54] Homma T., Fujiwara H., Osaki T., Fujii S., Fujii J. (2022). Consequences of a peroxiredoxin 4 (Prdx4) deficiency on learning and memory in mice. Biochem. Biophys. Res. Commun..

[55] Amatya B., Yang S., Yu P., Vaz de Castro P.A.S., Armando I., Zeng C., Felder R.A., Asico L.D., Jose P.A., Lee H. (2023). Peroxiredoxin-4 and Dopamine D5 Receptor Interact to Reduce Oxidative Stress and Inflammation in the Kidney. Antioxid. Redox Signal..

[56] Fujii J., Ochi H., Yamada S. (2025). A comprehensive review of peroxiredoxin 4, a redox protein evolved in oxidative protein folding coupled with hydrogen peroxide detoxification. Free Radic. Biol. Med..

[57] Tavender T.J., Bulleid N.J. (2010). Peroxiredoxin IV protects cells from oxidative stress by removing H_2_O_2_ produced during disulphide formation. J. Cell Sci..

[58] Kim D.Y., Choi B.Y. (2019). Costunolide—A Bioactive Sesquiterpene Lactone with Diverse Therapeutic Potential. Int. J. Mol. Sci..

[59] Huang X., Li H., Lu H., Aisa H.A., Li J. (2025). Chlorinated Guaiane-Type Sesquiterpene Lactones of Natural Origin. J. Nat. Prod..

[60] Barrera P., Sülsen V.P., Lozano E., Rivera M., Beer M.F., Tonn C., Martino V.S., Sosa M.A. (2013). Natural Sesquiterpene Lactones Induce Oxidative Stress in *Leishmania mexicana*. Evid.-Based Complement. Altern. Med..

[61] Li J., Zheng M., Xu Y., Yang X., Kang J. (2023). Target proteins profiling of irreversible kinase inhibitor pelitinib and discovery of degradation of PRDX4 by label free chemoproteomics. J. Pharm. Biomed. Anal..

[62] Zhou J.W., Bai Y., Guo J.Q., Li Y.Y., Liu Y.F., Liang C., Xing Y.R., Guo H.L., Qi T.X., Wu J. (2025). Peroxiredoxin 4 as a switch regulating PTEN/AKT axis in alveolar macrophages activation. Signal Transduct. Target. Ther..

